# A bioimage informatics platform for high-throughput embryo phenotyping

**DOI:** 10.1093/bib/bbw101

**Published:** 2016-10-14

**Authors:** James M Brown, Neil R Horner, Thomas N Lawson, Tanja Fiegel, Simon Greenaway, Hugh Morgan, Natalie Ring, Luis Santos, Duncan Sneddon, Lydia Teboul, Jennifer Vibert, Gagarine Yaikhom, Henrik Westerberg, Ann-Marie Mallon

**Affiliations:** MRC Harwell Institute, Harwell Campus, Oxfordshire

**Keywords:** bioimage informatics, image processing, embryonic phenotyping, automated analysis, high-throughput, software tools

## Abstract

High-throughput phenotyping is a cornerstone of numerous functional genomics projects. In recent years, imaging screens have become increasingly important in understanding gene–phenotype relationships in studies of cells, tissues and whole organisms. Three-dimensional (3D) imaging has risen to prominence in the field of developmental biology for its ability to capture whole embryo morphology and gene expression, as exemplified by the International Mouse Phenotyping Consortium (IMPC). Large volumes of image data are being acquired by multiple institutions around the world that encompass a range of modalities, proprietary software and metadata. To facilitate robust downstream analysis, images and metadata must be standardized to account for these differences. As an open scientific enterprise, making the data readily accessible is essential so that members of biomedical and clinical research communities can study the images for themselves without the need for highly specialized software or technical expertise. In this article, we present a platform of software tools that facilitate the upload, analysis and dissemination of 3D images for the IMPC. Over 750 reconstructions from 80 embryonic lethal and subviable lines have been captured to date, all of which are openly accessible at mousephenotype.org. Although designed for the IMPC, all software is available under an open-source licence for others to use and develop further. Ongoing developments aim to increase throughput and improve the analysis and dissemination of image data. Furthermore, we aim to ensure that images are searchable so that users can locate relevant images associated with genes, phenotypes or human diseases of interest.

## Introduction

High-throughput phenotyping is instrumental to the goals of the International Mouse Phenotyping Consortium (IMPC), a global endeavour to systematically produce and characterize a knockout mouse for every gene in the mouse genome [1]. Over the past 5 years, data for >3800 genes have been captured by 10 institutions around the world, adhering to a standardized pipeline of phenotyping procedures and modalities. A key element in the success of this project is the standardization of data to facilitate reliable and reproducible downstream analysis. One of the newest and richest sources of data within the IMPC is three-dimensional (3D) imaging for embryonic phenotyping.

Large-scale phenotyping projects such as the IMPC have benefited considerably from advances in biomedical imaging and computer vision. Applications span a broad range of modalities and model organisms, from time-lapse microscopy of mammalian cell lines [2] to multispectral imaging of plants [3]. In developmental biology, 3D imaging has been indispensable for understanding gene function in whole embryos at several developmental stages [4]. Within the IMPC, it is predicted that approximately one-third of knockout lines will be embryonic or perinatal lethal or subviable, which has driven the development of a tiered embryonic phenotyping pipeline [5]. In many cases, gross inspection alone does not reveal the cause of lethality and requires more in-depth interrogation by means of 3D imaging. Currently, embryo anatomy is captured using x-ray micro-computed tomography (micro-CT) [6], optical projection tomography (OPT) [7] and high-resolution episcopic microscopy (HREM) [8]. Automated image analysis methods have been adopted by the IMPC to quantify differences between wild-type and mutant populations using groupwise image registration [9, 10]. Recently, our efforts have been focused on managing the large volumes of data being submitted, improving the automated analysis and dissemination of results to the wider scientific community.

Imaging informatics first emerged following the advent of digital imaging in radiology, fulfilling the need to effectively manage large volumes of image data and metadata [11]. The field has since expanded into research environments to accommodate a diverse range of imaging platforms and data types [12]. The Open Microscopy Environment (OME) was designed to manage images of biological samples acquired by microscopy in a modality-agnostic fashion [13]. Image standardization is handled by Bio-Formats software, which is currently able to convert >140 image formats into the common OME-TIFF standard format. Images are linked to metadata using an extensible data model based on Extensible Markup Language (XML), and made available to users via the OMERO client-server platform [14]. Although 3D images are supported, there is limited provision for visualization that allows users to explore, compare and interact with the images. Furthermore, image overlays such as those provided by atlas-based registration and morphometry are not currently supported.

Of the various platforms developed for 3D imaging of embryonic development, the e-Mouse Atlas Project is among the best established [15]. The web-based interactive atlas provides images of embryos at native resolution, in the form of histological sections or full 3D reconstructions (e.g. magnetic resonance imaging). Images may be arbitrarily re-sliced and delivered to users as image tiles using the IIP3D protocol [16]. Similarly, the Deciphering Mechanisms of Developmental Disorders consortium has developed a portal, which provides HREM images of wild-type and mutant embryos using a tile-based image stack viewer [17]. Manual annotations of phenotypes are displayed as points of interest within the viewer, which are searchable as Mammalian Phenotype terms. In addition, pre-rendered movies of embryos in two and three dimensions are available. The Allen Brain Atlas provides comprehensive gene expression data from *in situ* hybridization at multiple developmental stages [18]. Users can search for genes or anatomical structures of interest and view gene expression profiles as high-resolution histological sections or pre-rendered 3D volumes.

Each of these portals provides new data in the form of releases, and so may remain relatively static for prolonged periods. The high-throughput nature of the IMPC requires a platform that can retrieve and disseminate images more frequently as and when they become available, while ensuring that data submitted by different institutions conform to the necessary standards for visualization and analysis. Although the full resolution image data are highly valued by domain experts, the desire to directly compare homologous structures in wild-type and mutant embryos in the web browser has warranted an approach that uses downsampled images. Nonetheless, by making the full resolution images available as downloads, it ensures that the full potential of these rich data sets can be realized. The task of deep interrogation and manual annotation may instead be tackled using desktop software that can fully harness the hardware resources available.

The IMPC embryonic phenotyping pipeline has presented several technical challenges that have led to the development of a bespoke bioimage informatics platform. In this article, we present a suite of software tools for the capture, processing, analysis, visualization and dissemination of 3D images. These tools have been used extensively in support of recent work on the discovery of novel developmental phenotypes, in which high-throughput 3D imaging has played an integral role [19]. Although primarily developed for the IMPC, all of the software is made available under an open-source licence to encourage wider adoption as standalone tools or as part of other 3D imaging pipelines.

## Background

Before the IMPC, data from large-scale phenotyping projects such as the European Mouse Disease Clinic had uncovered that between 30 and 40% of homozygous knockout lines were embryonic or perinatal lethal [20]. In addition to the already proposed plans to phenotype adult heterozygous mice, the IMPC adopted an additional phenotyping screen to capture viability and morphology data throughout mouse embryonic development to determine the window and cause of lethality. Following discussions among members of the consortium, it was unanimously decided that 3D imaging would serve as a core component of the pipeline [5].

A primary viability screen is performed by all centres on each new knockout line by monitoring heterozygous adult breeding patterns. A line is considered lethal if there are no viable homozygous pups at postnatal day 14, or subviable if there are <50% of the expected number of homozygous pups at weaning (according to normal Mendelian ratios). In addition to the primary viability screen, five phenotyping centres currently produce data for the embryonic and perinatal lethal phenotyping pipeline. A series of secondary viability screens is performed to establish the window of lethality. The key developmental stages are E9.5, E12.5, E14.5–E15.5 and E18.5 days post coitum (dpc). Gross morphological defects are identified by centres through visual inspection at each stage, such as abnormal limb development or craniofacial abnormalities. In addition to identifying potential abnormalities, this process helps to establish whether it is necessary to carry out additional imaging procedures. OPT has been adopted for imaging embryos at E9.5, whereas x-ray micro-CT is used for E14.5, E15.5 and E18.5. Other centre-specific imaging includes HREM at E14.5.

**Figure 1:**
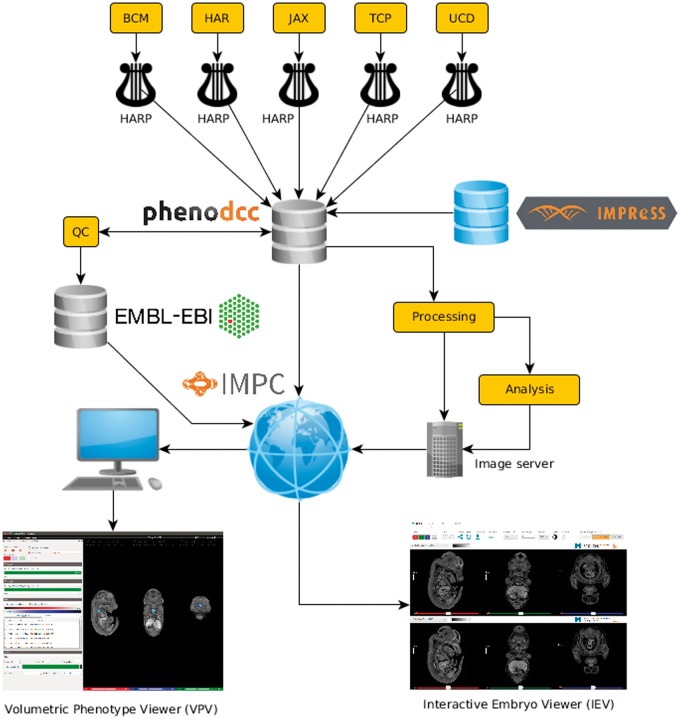
The IMPC informatics platform. Following acquisition and reconstruction, image data are processed to reduce file size and converted to a standard format. Specimen and experiment data are downloaded at the DCC (PhenoDCC), ensuring they adhere to the standard operating procedures specified in IMPReSS (https://www.mousephenotype.org/impress/). Three-dimensional image data are downloaded separately, and undergo additional processing before being made available for display on the IMPC portal [http://www.mousephenotype.org/data/search/gene?fq=(embryo_data_available:%22true%22)]. If sufficient data are available for a knockout line, automated image analysis [1] is performed, and the results are made displayable in the web-based viewer. All data are open access and freely downloadable at full resolution for desktop visualization. Quality control of images and analysis results is conducted before export to the CDA for long-term storage.

The data flow diagram in Figure 1 shows the process by which 3D image data are captured and fed through the IMPC informatics platform. The task of collecting, curating, analysing and disseminating phenotyping data within the IMPC is the joint responsibility of the Data Coordination Centre (DCC) at MRC Harwell Institute, and the Core Data Archive (CDA) at the EMBL European Bioinformatics Institute. Specimen and experiment data are provided by IMPC centres as XML files, which are uploaded to their respective file transfer protocol sites, and retrieved by the DCC on a daily basis. Following validation, the data are inserted into a MySQL database. Any associated media files are retrieved separately using a dedicated media downloader, adhering to a hierarchical directory structure. This enables easy retrieval of image files according to centre, gene and phenotyping procedure [21, 22].

**Figure 2:**
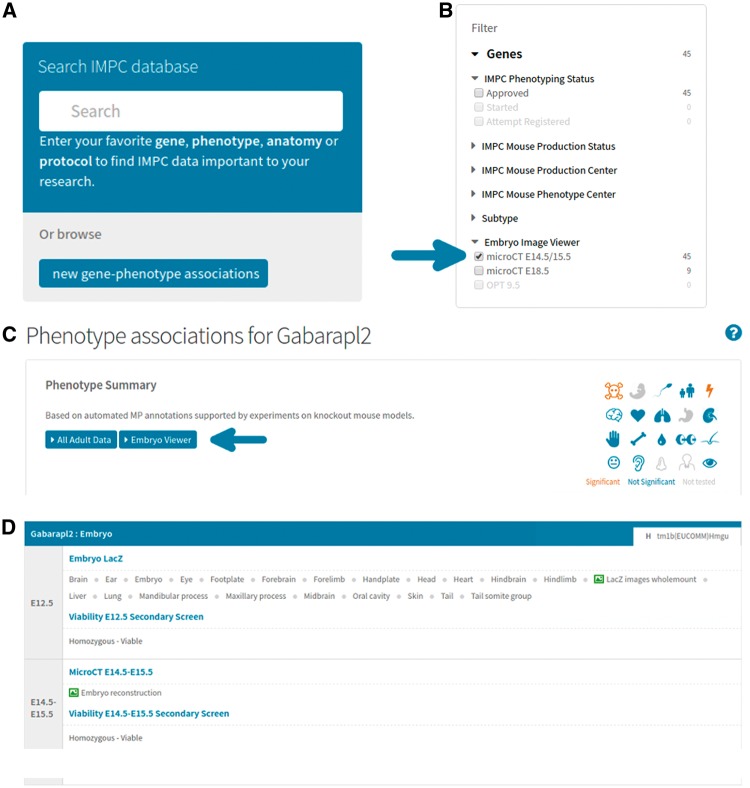
Accessing 3D image data on the portal. (**A**) From mousephenotype.org, users can search for genes or phenotypes of interest in the search box. (**B**) On the search results page, users may then filter the list of genes returned to only those with 3D images available. (**C**) On selecting a gene of interest (e.g. http://www.mousephenotype.org/data/genes/MGI:1890602), the user will be able to view all phenotyping data associated with that gene. Clicking the ‘Embryo Viewer’ button will display the 3D image data. (**D**) Users can also view data from embryo-specific procedures on the same page, such as viability and lacZ expression (http://www.mousephenotype.org/data/genes/MGI:1890602#heatmap).

As with all data exported to the DCC, image files and their associated metadata are submitted in accordance with the standard operating procedures outlined in International Mouse Phenotyping Resource of Standardised Screens (IMPReSS) (https://www.mousephenotype.org/impress). Within IMPReSS, an individual procedure (e.g. E18.5 micro-CT) describes an assay or collection of assays that may be used to measure or score phenotypes within a biological system or process. Each procedure comprises a number of parameters (e.g. x-ray source voltage), which are the actual measurements, images or metadata that are collected as part of the assay. This approach is an effective solution to the problem of standardization, ensuring that participating centres adhere to previously agreed on protocols that can be computationally validated before insertion into a database. Collection of data in its rawest form (including associated metadata) is imperative to prevent inconsistencies in the subsequent analyses. For 3D imaging, all images are submitted at their original resolutions alongside metadata that include sample preparation details, equipment specifications and acquisition parameters. Once downloaded at the DCC, 3D images undergo additional processing to facilitate automated image analysis and visualization on the IMPC portal. Figure 2 describes the process by which users can find and view 3D image data.

## Methods and technologies

In developing the infrastructure for the 3D imaging pipeline, a number of existing technologies were considered to minimize lead times and ensure that data could be captured and disseminated at the earliest opportunity. In this section, we describe the various tools and their underlying technologies that have been used to capture, validate, analyse and disseminate 3D image data.

### Image processing and standardization

**Figure 3:**
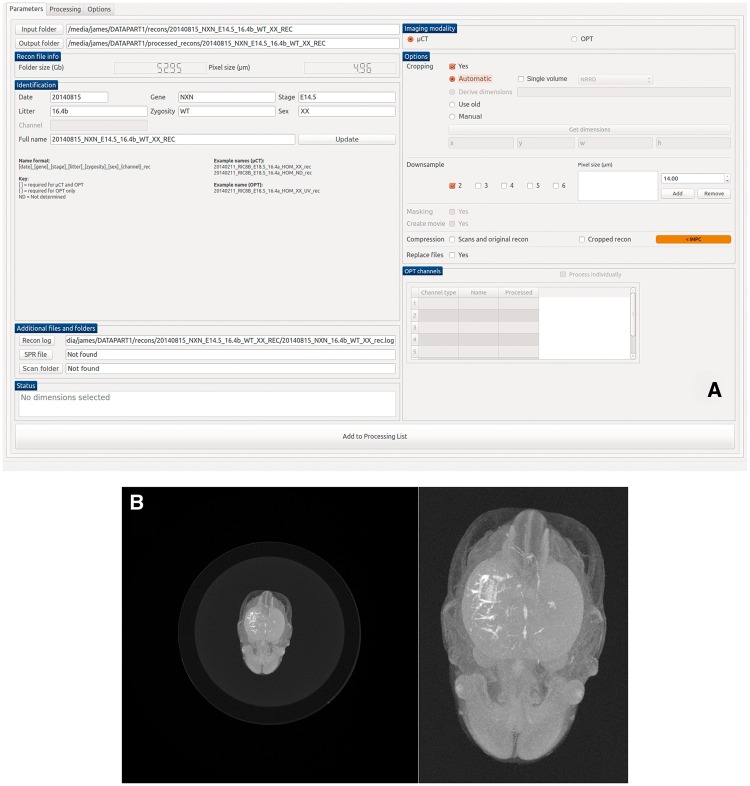
HARP is a desktop application for Windows and Linux. (**A**) The main application window. In the left panel, users can configure output directories and metadata. In the right panel, options for cropping, resizing and compression are provided. (**B**) A maximum intensity projection of a micro-CT image before (left) and after (right) automatic cropping. Here, the cropping process decreases the total file size from 56.8 to 4.5 GB (uncompressed). Image compression further reduces the file size to 3.6 GB, drastically reducing storage requirements.

Although all procedures are standardized wherever possible, there are inevitable differences both within and between institutions in terms of instrumentation, sample preparation and post-processing. Furthermore, extraneous voxels in high-resolution 3D images (i.e. empty space) lengthen the time taken to process and upload. To accommodate these issues, bespoke image processing software has been developed by the IMPC that standardizes image data before submission to the DCC. The Harwell Automated Reconstruction Processor (HARP) is an open-source, cross-platform application developed in Python (Figure 3A). It is designed to operate on 3D image data represented as two-dimensional (2D) slices in a variety of formats (tiff, bmp, jpg, etc.). Although it was primarily designed for tomographic images of embryos, it can in practice be used for other imaging modalities, specimen types and fields of study. The standard output format used by HARP is Nearly Raw Raster Data (NRRD). The latest version of the software can be downloaded from GitHub (https://github.com/mpi2/HARP).

HARP provides options to automatically crop image volumes to remove any undesirable empty space around the specimens. This is achieved by performing an adaptive maximum intensity projection on the image data, followed by an Otsu threshold, from which a bounding box is calculated (Figure 3B). In instances where an automated crop is unsuitable, users can manually draw a bounding box on the projection image. For modalities where multiple channels are collected (e.g. OPT), the same crop box can be automatically applied to all available channels. Images can also be compressed using bzip2 compression to minimize bandwidth usage during download. With images being captured at a variety of resolutions, HARP also provides image resizing by integer factors (binning) or to arbitrary pixel sizes (resampling). Individual processing jobs are appended to a queue and executed in sequence, such that multiple jobs can be set up and left running overnight. User interaction is minimized further by automatically creating output directory structures and notifying users of any image data that have already been processed.

The software was developed for users wishing to automate simple processing of images from a wide range of 3D imaging modalities. For projects where large numbers of images are required by one or more individuals, HARP also helps to establish a consistent and appropriate file name convention (date, allele, zygosity, sex, etc.) for ease of retrieval and archival. Minimal experience is required to use the software, and a detailed user guide is provided for reference.

### Automated image analysis

Three dimensional imaging modalities such as micro-CT and OPT produce a wealth of information that can be extremely time-consuming to manually annotate, and requires expert knowledge to correctly identify abnormal phenotypes. Manual annotation is a bottleneck within large-scale projects such as the IMPC, where large numbers of image volumes are submitted, staff numbers with expert knowledge may be limited and rapid dissemination of the submitted images to the public is paramount. Manual annotation of 3D images is also essentially a qualitative exercise, where regions identified as abnormal by a biologist are assigned a descriptive term. Coverage is often restricted to the specific expertise of the operator, resulting in biases that do not reflect the full extent of the phenotypic landscape. Even if enough resources are available to thoroughly annotate images, subtle phenotypes can be missed by even the most experienced operators. Automated analysis has the potential to drastically decrease the time required to analyse these images, and provide highly informative, new information in a systematic, unbiased, quantitative and statistically robust fashion.

The IMPC has adopted the automated phenotyping pipeline developed at the Mouse Imaging Centre (MICe) in Toronto for the analysis of homozygous lethal embryos imaged by micro-CT [10]. The underlying principle of the method relies on comparing wild-type and mutant embryos in a common coordinate space. To directly compare homologous structures between groups, images are brought into spatial alignment using groupwise image registration. Morphological differences between the populations are then established through statistical analysis of the deformation fields generated during registration. Additionally, differences in image intensity because of highly dysmorphic or absent structures may be identified. The results of the analysis come in the form of 3D quantitative heatmaps, which when overlaid with the embryos reveal statistically significant regions of dysmorphology as coloured ‘blobs’. Registration of a labelled atlas with the population average (mean of all registered images) also allows for total organ volume calculations to be performed, allowing for differences in overall organ size to be determined.

Following the successful application of the software to a number of embryonic lethal lines, we have sought to address several practical questions on how the analysis could be deployed at the DCC. Additionally, there has been a strong interest from consortium members to analyse their own data locally, without the need for a high specification grid. To this end, a lightweight alternative to the MICe pipeline has been developed at the DCC for use by the wider scientific community on relatively low specification machines, with potential applicability to other model organisms and tissues (manuscript in preparation).

### Image segmentation

Automated image segmentation is a highly desirable feature of the automated analysis pipeline, as it makes differences in total organ volume readily calculable. Using the existing method, this is achieved by first manually segmenting an average embryo to create an atlas. Following registration of the atlas towards a combined average of wild-type and mutant embryos, image labels may be propagated by applying the respective inverse transformations to the atlas labels [23]. In the original work, segmentation of the E15.5 average into 48 anatomical structures was an entirely manual process that took >400 h [9]. In a high-throughput context, this becomes prohibitively time-consuming, as new atlases will need to be produced for each phenotyping centre and developmental stage. To accommodate this limitation, a semi-automated method has been devised to minimize the effort needed to produce a sufficiently accurate segmentation in a substantially shorter time.

**Figure 4:**
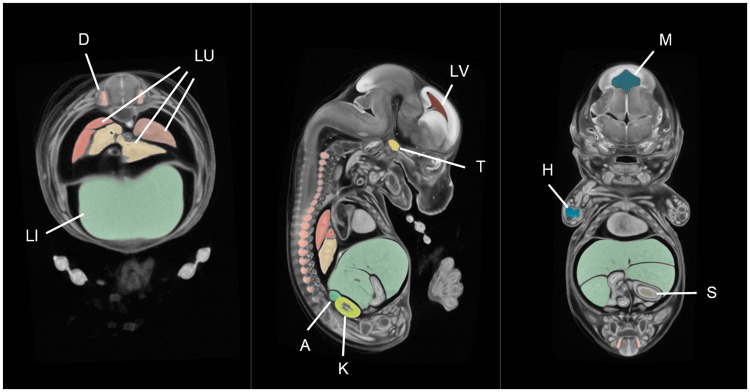
A mouse embryo atlas at E14.5 dpc. This atlas is an average of 16 micro-CT images of C57Bl/6N (wild-type) embryos produced using in-house software. The average was segmented in 3D Slicer using our WASP plug-in, taking ∼8 h in total. The organs shown in this figure include the liver (LI), lungs (LU), dorsal root ganglia (D), trigeminal ganglion (T), humerus primordium (H), kidney (K), adrenal gland (A), stomach lumen (S) lateral brain ventricle (LV) and mesencephalic vesicle (M).

One of the main benefits of image averaging is the increased signal-to-noise ratio. As a consequence, organ boundaries with increased saliency make image segmentation highly amenable to semi-automated methods. With this in mind, a tool for 3D embryo segmentation has been developed by the IMPC as a plug-in for 3D Slicer called the Watershed Annotation and Segmentation Plugin (WASP). The software works by producing a series of imperfect candidate segmentations using the watershed algorithm [24]. Once complete, users can annotate the candidate segmentations to build up a single label map consisting of only the structures of interest. For an experienced user, an E14.5 average embryo can be segmented into ∼30 structures in a single working day. To improve label accuracy, some additional manual curation is typically performed using ITK-SNAP [25]. An example E14.5 atlas is shown in Figure 4. In addition to the creation of atlases, WASP can be used to segment other model organisms and image types.

WASP was designed primarily for those with experience using 3D Slicer or similar software who wished to segment micro-CT images of embryos or other biological specimens with sufficient contrast. For those who are unfamiliar with the software, an instructional video and documentation are provided at http://wiki.slicer.org/slicerWiki/index.php/Documentation/4.5/Extensions/Wasp.

## Visualization and dissemination

One of the core principles of the IMPC is that the data are open and accessible to all. Following successful upload to the DCC, users can view and download phenotyping data for individual genes from their respective gene pages (An example gene page for Tead1: http://www.mousephenotype.org/data/genes/MGI:101876). One of the key limitations of the web portal up until recently was the inability to access and interpret 3D imaging data and automated analysis results. This is now made possible using bespoke software for the desktop and web browser.

### Volume phenotype viewer

The output from the automated phenotyping pipeline (MICe) consists of *t*-statistic heatmaps that are overlaid with the population average to reveal statistically significant differences. These images are currently viewed using software called MNI-display, developed at the McConnell Brain Imaging Centre [26]. However, there are several manual steps required to load the data from a single analysis, which can be somewhat cumbersome to perform for an inexperienced user. Software such as 3D Slicer provides a wealth of features that allow for the data to be more easily viewed and manipulated, but does not natively support image files in MINC format, as used by the MICe pipeline. Furthermore, neither of these tools have the ability to display vector fields, which are useful in analysing registration results.

**Figure 5:**
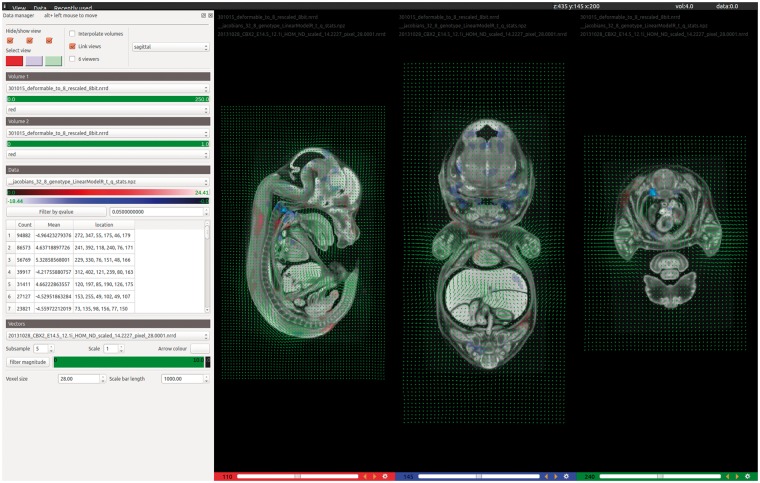
VPV is a desktop application for visualizing the results of 3D image registration. Designed to accompany the automated analysis pipeline, VPV brings together many of the most desirable features found across existing visualization tools into a simple, intuitive graphical user interface. The screenshot shows the software being used to view the results of analysing Cbx2 knockout embryos imaged by micro-CT. Users can simply drag and drop their images and analyse data to instantly view regions of significant dysmorphology as heatmaps, shown in red/blue overlays. Deformation (vector) fields can also be overlaid to observe how the registration has behaved, as shown in green.

To overcome these limitations, we have developed a slice-based volume phenotype viewer (VPV) for the desktop that enables rapid identification of phenotypically abnormal structures (Figure 5). Data are easily loaded by drag and drop, with support for most commonly used file formats. On loading the registration results, embryos may be overlaid with the *t*-statistic heatmaps to reveal regions of dysmorphology. We have adopted a hot red/blue colour scheme to be consistent with previously reported results. The heatmap data can be filtered by *t*-statistic value to emphasize regions of different statistical significances. Vector field data can also be loaded into VPV and filtered by magnitude to identify where the most significant deformations have taken place during registration.

Subtle phenotypes may only be represented by small regions within the *t*-statistic overlays, which can be difficult to locate by eye. Therefore, during loading, VPV calculates connected regions of non-zero voxels throughout the heatmap volume. These are then reported in a table that displays the position of each significant region, its volume in voxels and the mean *t*-statistic value. Clicking on a table entry takes the user directly to the midpoint of the region and identifies it with a bounding box. Deformation fields generated during the registration can also be viewed at configurable scales and granularity, with the option to filter by magnitude. This allows users to rapidly identify regions in the volume that had to move significantly during registration towards the target. VPV supports the overlay of multiple images, heatmaps and vector fields simultaneously if required. Other features include customizable scale/colour bars for figure generation, image interpolation and up to six independently configurable image viewers. VPV can be downloaded for free from GitHub (https://github.com/mpi2/vpv), and includes a Windows installer.

VPV was initially designed as a lightweight visualization tool to perform basic functions for interrogating 3D images and overlays. The software has since become a powerful asset for optimizing image registration parameters and interpreting the results of the automated analysis. As such, the software is primarily geared towards a more technical user base such as those from medical image computing communities.

### Interactive embryo viewer

**Figure 6:**
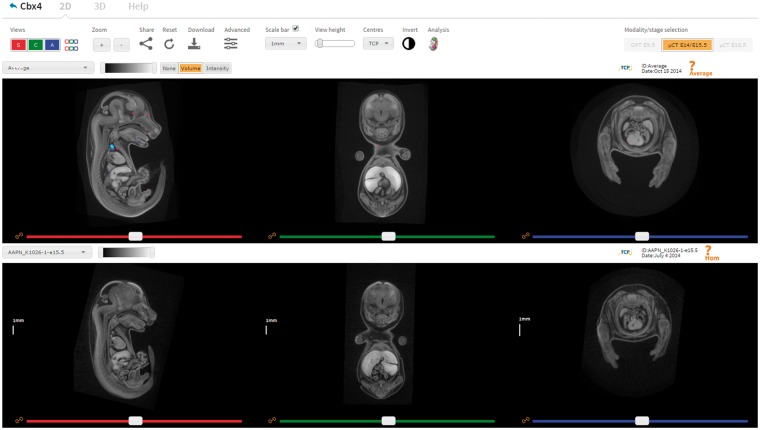
IEV is a web-based tool that allows users to directly compare wild-type and mutant embryos in a dynamic, intuitive manner. Users can navigate the volumes in synchrony by using the sliders, mouse wheel or crosshair tool (shift key while moving the mouse). The settings panel provides options to toggle viewports on/off, configure the scale bar, zoom in/out, create bookmarks and switch modality. Each viewer has its own drop-down box listing the embryos available to view, an image contrast slider, and the ability to link/unlink individual viewports. Other features include fully interactive 3D renderings, ability to bookmark viewer state and share them and full resolution downloads. Automated analysis results can also be displayed where available, as shown here in the top viewer for the gene *Cbx4* (http://www.mousephenotype.org/embryoviewer/?mgi=MGI:1195985&wn=Average). Images and analysis results were produced by The Centre for Phenogenomics, Toronto. A user guide can be viewed and downloaded by clicking the Help tab.

In addition to the desktop image viewer VPV, a web-based tool for viewing 3D image data has been developed and integrated into the IMPC portal (Figure 6). The Interactive Embryo Viewer (IEV) is a comparative tool that provides many of the features typically found in fully fledged desktop viewers. Developed using the X Toolkit [27], downsampled images are downloaded in full as compressed NRRD files (∼10 Mb), and made displayable in the web browser as 2D slices or 3D renderings. Alternatively, the volumes may be downloaded at native resolution and viewed in VPV or other freely available software such as Fiji and 3D Slicer. At the time of publication, over 1 TB of micro-CT and OPT image data have been imported for 80 knockout lines. Image data and overlays representing the results of automated analysis are downloadable from within IEV by clicking the Download button.

For any given gene of interest, IEV will retrieve all available image data for users to visualize and interact with. For the purpose of comparison, any wild-type controls submitted by the same phenotyping centre are also provided. Wild-type images are shown in the top viewer by default, with mutants shown in the bottom viewer. On the top left of each viewer, volumes can be selected via drop-down boxes. Associated metadata, such as scan date, is displayed on the top right of each viewer. Individual volumes are made navigable via three orthogonal viewports, each controlled by a separate slider. Users can explore and compare images of mutant embryos with their wild-type counterparts by linking corresponding viewports as desired, allowing for homologous anatomical structures to be studied side by side. Viewports can be dynamically updated according to mouse position to localize anatomical structures in all three dimensions. Configurable scale bars are also provided and can be placed at arbitrary locations on each viewport for simple measurements. Viewers can also be resized and transposed to suit different screen aspect ratios.

In some instances, images from the same centre may vary in terms of image intensity. Sliders are provided in both 2D and 3D mode to restrict image intensities to a narrower dynamic range, which is typically used to reduce image noise and increase overall brightness. In addition, the entire colour map may be inverted such that low-intensity pixels appear white, and high-intensity pixels appear black. Images can also be zoomed in and out using the zoom buttons or mouse. Having generated a view that highlights a phenotype of interest, clicking the Share button provides a Uniform Resource Locator (URL) that encodes the user’s settings. Almost all configurable features of IEV can be preserved and reproduced by visiting the URL in any supported browser. This feature is currently used to share image data with the scientific community via the IMPC Twitter (@impc) page on a monthly basis. In addition to the 2D slice viewers, entire embryos may also be viewed and manipulated in three dimensions as volume renderings. Wild-type and mutant embryos are displayed side by side, allowing them to be arbitrarily rotated, zoomed and panned using the mouse.

The results output by the automated analysis pipeline are made viewable in IEV as heatmap overlays, and are accessible by clicking the Analysis button in the control panel. This will display the ‘population average’ in the top viewer overlaid with the heatmap results. Buttons are displayed above the viewer that allow users to switch between the volume and intensity heatmaps, or to overlay segmented organ labels. Currently, analysis data for two knockout lines (*Cbx4* and *Eya4*) are available for display in IEV. To our knowledge, this is the first time that heatmaps derived from tensor- and voxel-based morphometry have been made available in the web browser in such a way that allows dynamic user interaction. Now, having fully established strategies for deploying the automated analysis software, routine analysis of lines with sufficient sample numbers will begin later this year (2016).

IEV was primarily designed for developmental biologists as an easy-to-use, intuitive web interface requiring minimal experience to make use of its core functions. A short user guide is provided to help users orientate themselves with the orthogonal viewports and functions, and a short demonstration video is provided in the Supplementary Information. It can be used on relatively low specification computers without any significant performance issues, and works in any modern browser with WebGL support. Although no thorough load testing has been conducted, the software has been demonstrated at several conferences and workshops with up to 25 simultaneous users without issue. We anticipate that as the user base grows, the image server will likely become a limiting factor in its usability, and so a cloud-based storage solution may be more appropriate in future.

### Comparison with existing platforms

The informatics platform developed for 3D imaging within the IMPC has presented several technical challenges that have been addressed through development of bespoke software tools. Although no existing platform to our knowledge provides all of the necessary functionality, there are several that have provided inspiration and ideas for future development. BioImageXD is a general-purpose, open-source platform for 3D image processing, analysis and visualization [28]. The basic functionality provided by the software is similar to that which is provided in separate desktop applications provided by the DCC, such as batch processing (HARP), image segmentation (WASP) and slice-based visualization (VPV). Having developed these software tools using the Python programming language (and having many software libraries in common), it would be relatively straightforward to merge them into a single desktop application. Most of the more sophisticated functionality provided by BioImageXD such as cell tracking are not required for our purposes.

Bisque is a web-based platform for managing biological images and quantitative analysis data [29]. Images and metadata are captured using flexible key–value pair model, allowing for arbitrary annotations of a variety of types that may be nested. This would be a highly desirable, as it would allow for images and analysis data to be indexed by, and searchable on, the IMPC portal. The basic functionality to capture such annotations has been implemented at the DCC, and will be made available in IEV in future. BisQue also provides a wide range of image analysis tools online, including the Matlab implementation of the watershed algorithm. Although WASP uses a similar implementation from the Insight Toolkit, it also provides the necessary annotation tools for atlas creation and mesh generation through integration with 3D Slicer.

The Virtual Brain Explorer for Zebrafish (ViBE-Z) is a framework for analysing gene expression in the brains of zebrafish larvae by mapping confocal microscopy data to an anatomical reference [30]. Conversion to standard format is performed using ImageJ, which was previously used by the IMPC until HARP was deployed to perform such tasks in batch. Data are then uploaded to the web and processed using the ViBE-Z software. Automated landmark annotation and elastic registration of images with a reference atlas take ∼30 min, after which users may download and view the data in ImageJ rather than a web-based viewer. The process of alignment allows for large-scale colocalization analyses across many genes to be performed. The automated analysis method used by the IMPC would be highly amenable to such an approach, using the existing landmark-free registration method for automated phenotype detection. Given sufficient data, this would allow for genes with similar phenotypic profiles in the developing embryo to be clustered and investigated.

## Future directions

HARP is now being used routinely by IMPC centres to process their 3D imaging data, enabling the upload of >750 image reconstructions to date. New features under development include a batch processing mode that would allow HARP to be run as a scheduled job to process new reconstructions as they emerge, without the need for manual invention. In addition, WASP will undergo further development to improve usability on multiple platforms.

Visualization of 3D imaging data and analysis results is now possible on the desktop or in the web browser. Future additions to VPV include functionality that would allow users to manually annotate regions of interest (ROI) in the mouse embryo with ontological terms. Standardization of spatial ROIs and their associated terms will enable centres to easily upload their manual annotations to the DCC, and make them readily viewable in IEV. Results from the automated analysis will also be associated with the same ontological terms for internal consistency.

In future, specific phenotypes will be searchable on the IMPC portal, bringing users directly to abnormal regions within the whole embryo that have been annotated either manually or automatically. An appropriate method for mapping anatomical phenotypes to a reference atlas is currently under development. In the coming months, IEV ‘lite’ will be made available as a standalone viewer that will allow institutions to host and display their own 3D image data (https://github.com/mpi2/iev). We are also exploring the possibility of integrating IEV into OMERO, allowing its users to directly compare their 3D image data in the web browser for the first time.

**Key Points**

- The high-throughput, multinational nature of the IMPC has necessitated a robust infrastructure to capture and analyse these data. Functional annotation of the mouse genome has relied heavily on data standardization to be successful.
- The role of 3D imaging in understanding disorders of embryonic development has presented a substantial technical challenge. Standardization of protocols and image formats has enabled large quantities of 3D imaging data to be handled efficiently.
- Automated analysis has proven invaluable to understanding gene function in the mouse embryo, but has required significant support from bespoke software to process, interpret and disseminate the 3D imaging data. Development of new tools has allowed users to fully harness the power of these new methodologies with minimal computational expertise.
- All tools are available to the broader imaging community, having been designed to function independently and without the need for databases or substantial computing power.

### Supplementary data

Supplementary data are available online at http://bib.oxfordjournals.org/.

## Supplementary Material

Supplementary Data

## Notes

**James Brown** is a postdoctoral researcher at MRC Harwell Institute. His research interests include statistical shape modelling, image registration and quantitative 3D image analysis for functional genomics.

## Notes

**Neil Horner** is a postdoctoral researcher at MRC Harwell Institute. His research interests lie in automated image analysis techniques to uncover abnormal mammalian phenotypes in tomographic image data.

## Notes

**Henrik Westerberg** is the team leader of bioimage informatics within BioComputing at MRC Harwell Institute. His research interests include automated annotation of phenotypes from a variety of imaging modalities and data sources, and the broader integration of these data with publicly available data sets worldwide.

## Notes

**Ann-Marie Mallon** is the head of BioComputing at MRC Harwell Institute. Her research focuses on three main areas: the management and analysis of large-scale mouse phenotyping data, the analysis of NGS and transcriptomics data and 3D systems imaging.
